# Bone health in women with premature ovarian insufficiency/early menopause: a 23-year longitudinal analysis

**DOI:** 10.1093/humrep/deae037

**Published:** 2024-02-23

**Authors:** A R Jones, J Enticott, P R Ebeling, G D Mishra, H T Teede, A J Vincent

**Affiliations:** Monash Centre for Health Research and Implementation, Monash University, Melbourne, VIC, Australia; Department of Endocrinology, Monash Health, Melbourne, VIC, Australia; Monash Centre for Health Research and Implementation, Monash University, Melbourne, VIC, Australia; Department of Endocrinology, Monash Health, Melbourne, VIC, Australia; Department of Medicine, School of Clinical Sciences, Monash University, Melbourne, VIC, Australia; Australian Women and Girls’ Health Research Centre, School of Public Health, University of Queensland, Brisbane, QLD, Australia; Monash Centre for Health Research and Implementation, Monash University, Melbourne, VIC, Australia; Department of Endocrinology, Monash Health, Melbourne, VIC, Australia; Monash Centre for Health Research and Implementation, Monash University, Melbourne, VIC, Australia; Department of Endocrinology, Monash Health, Melbourne, VIC, Australia

**Keywords:** premature ovarian insufficiency, early menopause, osteoporosis, fractures, longitudinal

## Abstract

**STUDY QUESTION:**

What is the frequency of, and predictors for, osteoporosis, fractures, and osteoporosis management (investigation, treatment) in women with premature ovarian insufficiency (POI; menopause <40 years) and early menopause (EM; menopause 40–44years)?

**SUMMARY ANSWER:**

Over the 23-year follow-up duration, at a mean age of 68 years, women with POI/EM had higher osteoporosis/fracture risk and prevalence, higher osteoporosis screening and anti-osteoporosis medication use compared to women with usual age menopause; increasing age was predictive of increased risk of osteoporosis/fracture and menopause hormone therapy (MHT) prior to or at study entry (aged 45–50 years) was protective.

**WHAT IS KNOWN ALREADY:**

Women with POI/EM have increased risk of osteoporosis and fractures with limited data regarding risk factors for reduced bone density and fractures. Clinical guidelines recommend screening with dual X-ray absorptiometry (DXA) and treatment with MHT for most women with POI/EM to reduce osteoporosis and fracture risk; however, studies indicate gaps in osteoporosis knowledge, guideline uptake, and management adherence by clinicians and women.

**STUDY DESIGN, SIZE, DURATION:**

The Australian Longitudinal Study on Women’s Health is a prospective longitudinal study of Australian women. This study uses the cohort of women born between 1946 and 1951, surveyed nine times between 1996 and 2019. Data from the Australian administrative health records, including hospital admissions data (fractures, osteoporosis), Medicare Benefits Schedule (DXA), and the Pharmaceutical Benefits Scheme (PBS; MHT, anti-osteoporosis medication, available only from 2002) were linked to survey data.

**PARTICIPANTS/MATERIALS, SETTING, METHODS:**

Survey respondents with self-reported age of menopause were included. POI/EM was defined as menopause <45 years. *T*-test or chi-square were used for comparisons at baseline (*P* < 0.05 indicates significance). Generalized estimating equations for panel data explored predictors for the longitudinal outcomes of osteoporosis, fractures, DXA rates, MHT use, and anti-osteoporosis medication (in women with osteoporosis/fracture, from Survey 4 onwards only). Univariable regression was performed, and variables retained where *P* < 0.2, to form the multivariable model, and bootstrapping with 100 repetitions at 95% sampling of the original dataset to ensure robustness of results.

**MAIN RESULTS AND THE ROLE OF CHANCE:**

Eight thousand six hundred and three women were included: 610 (7.1%) with POI/EM. Mean (SD) baseline age was 47.6 (1.45) years in the entire cohort and mean (SD) age of menopause was 38.2 (7.95) and 51.3 (3.04) years in women with POI/EM and usual age menopause, respectively (*P* < 0.001). Over the 23 years, of women with POI/EM, 303 (49.7%) had osteoporosis/fractures, 421 (69.0%) had DXA screening, 474 ever used MHT (77.7%), and 116 (39.1%) of those with osteoporosis/fractures used anti-osteoporosis medication. Of women with usual age menopause, 2929 (36.6%) had osteoporosis/fractures, 4920 (61.6%) had DXA screening, 4014 (50.2%) used MHT, and 964 (33.0%) of those with osteoporosis/fractures used anti-osteoporosis medication. Compared to women with menopause at age ≥45 years and after adjusting for other risk factors, women with POI/EM had increased risk of osteoporosis (odds ratio [OR] 1.37; 95% CI 1.07–1.77), fractures (OR 1.45; 1.15–1.81), DXA testing (OR 1.64; 1.42–1.90), MHT use (OR 6.87; 5.68–8.30), and anti-osteoporosis medication use (OR 1.50; 1.14–1.98). In women with POI/EM women, increasing age was associated with greater risk of osteoporosis/fracture (OR 1.09; 1.08–1.11), and MHT prior to or at study entry (aged 45–50 years), was protective (OR 0.65, 0.45–0.96). In women with POI/EM, age (OR 1.11; 1.10–1.12), fractures (OR 1.80, 1.38–2.34), current smoking (OR 0.60; 0.43–0.86), and inner (OR 0.68; 0.53–0.88) or outer regional (OR 0.63; 0.46–0.87) residential location were associated with DXA screening. In women with POI/EM, increasing age (OR 1.02; 1.01–1.02), and currently consuming alcohol (OR 1.17; 1.06–1.28), was associated with having ever used MHT. In the 299 women with POI/EM and osteoporosis/fractures, only 39.1% ever received treatment with an anti-osteoporosis medication. Increasing age (OR 1.07; 1.04–1.09) and lower BMI (OR 0.95; 0.92–0.98) were associated with greater likelihood of treatment with anti-osteoporosis medication.

**LIMITATIONS, REASONS FOR CAUTION:**

Survey data including age of menopause were self-reported by participants; fracture questions were not included in the 2001 survey, and location or level of trauma of self-reported fractures was not asked. Additional risk/protective factors such as vitamin D status, calcium intake, and exercise were not able to be included. Due to sample size, POI and EM were combined for all analyses, and we were unable to differentiate between causes of POI/EM. PBS data were only available from 2004, and hospital admissions data were state-based, with all of Australia were only available from 2007.

**WIDER IMPLICATIONS OF THE FINDINGS:**

This study supports previous literature indicating increased risk of osteoporosis and fractures in women with POI, and adds evidence for women with POI/EM, where there was a relative paucity of data. This is the first study to analyse a variety of clinical and demographic risk factors for osteoporosis and fractures in women with POI/EM, as well as analysing investigation and treatment rates. In these women, using MHT prior to or at study entry, aged 45–50 years, was protective for osteoporosis/fractures; however, having ever used MHT was not, highlighting the importance of early treatment with MHT in these women to preserve bone strength. Although women with POI/EM and osteoporosis or fractures were more likely to use anti-osteoporosis medications than those with usual age menopause, overall treatment rates are low at <40%, demonstrating a significant treatment gap that should be addressed to reduce future fracture risk.

**STUDY FUNDING/COMPETING INTEREST(S):**

This study was funded by The Australian NHMRC Centre of Research Excellence Women’s Health in Reproductive Life (CRE-WHIRL, project number APP1171592). A.R.J. is the recipient of a National Health and Medical Research Council post-graduate research scholarship (grant number 1169192). P.R.E. is supported by a National Health and Medical Research Council grant 1197958. P.R.E. reports grants paid to their institution from Amgen, Sanofi, and Alexion, honoraria from Amgen paid to their institution, and honoraria from Alexion and Kyowa-Kirin.

**TRIAL REGISTRATION NUMBER:**

N/A.

## Introduction

The usual age of menopause globally is between 48 and 52 years (InterLACE Study Team, 2019). Premature ovarian insufficiency (POI), loss of ovarian function occurring before the age of 40 years, affects around 2–4% of women, and early menopause (EM), menopause occurring between 40 and 45 years, affects up to 12% of women (Mishra *et al.*, 2017; Golezar *et al.*, 2019). EM can occur spontaneously, or secondary to medical or surgical therapies such as bilateral oophorectomy, radiotherapy, or chemotherapy (The ESHRE Guideline Group on POI *et al.*, 2016).

Long-term health concerns associated with POI/EM include increase in cardiovascular disease, cognitive dysfunction, and reduced bone mineral density (BMD), as well as reduced overall quality of life and life expectancy, largely due to cardiovascular disease (Muka *et al.*, 2016; The ESHRE Guideline Group on POI *et al.*, 2016).

Peak bone mass is usually achieved during the third decade of life, and oestrogen deficiency during childhood to young adulthood can prevent optimal peak bone mass accrual (Biller *et al.*, 1989; Weaver *et al.*, 2016; Siegel *et al.*, 2017). Bone mass remains relatively stable during mid-life, then, during the late perimenopause and postmenopausal periods, there is rapid loss of bone density, of around 2% per year (Rosenthal *et al.*, 1989; Ahlborg *et al.*, 2003; Finkelstein *et al.*, 2008). Depending on the age of menopause, women with POI/EM may experience both lower peak bone mass, and earlier menopausal bone loss, predisposing to osteoporosis and fractures (Nguyen *et al.*, 2021).

Studies consistently demonstrate lower BMD in women with POI, with a corresponding increase in fracture risk (Sullivan *et al.*, 2015, 2017; Shea *et al.*, 2021; Costa *et al.*, 2023). Risk factors for lower BMD in women with POI include lower age of menopause, low vitamin D, low body weight, lack of exercise, delay in diagnosis, poor adherence to menopause hormone therapy (MHT), and low calcium intake (Popat *et al.*, 2009). Despite affecting significantly more women than POI alone, there is a relative paucity of data on bone health in women with POI/EM, and studies examining osteoporosis and fracture risk in these women have had mixed results, with some showing increased risk (Hadjidakis *et al.*, 2003; Paganini-Hill *et al.*, 2005; Anagnostis *et al.*, 2019) and others showing no difference (Papaioannou *et al.*, 2005; Banks *et al.*, 2009).

International guidelines recommend the use of MHT in women with POI/EM, until the usual age of menopause (The ESHRE Guideline Group on POI *et al.*, 2016; The North American Menopause Society, 2022). MHT has been shown to improve BMD in these women, and, although evidence for fracture reduction in this population is unclear, data extrapolated from studies in postmenopausal women in general has shown significant fracture reduction (Burgos *et al.*, 2017; Sullivan *et al.*, 2017; Barrionuevo *et al.*, 2019; Costa *et al.*, 2023). However, data suggest that many women fail to receive appropriate MHT to protect their bones (Bachelot *et al.*, 2016; Goh *et al.*, 2019).

This study aimed to investigate the prevalence of osteoporosis and fractures in women with POI/EM and the risk factors for developing osteoporosis and fractures, as well as examining gaps in osteoporosis investigation and treatment in a country with universal healthcare and subsidized access to medication.

## Materials and methods

### Participants

The Australian Longitudinal Study on Women’s Health (ALSWH) is an ongoing prospective study examining the health of Australian women. Full details of the study are described elsewhere (Brown *et al.*, 1996). In brief, four birth cohorts of women were invited to participate, with random selection from the Australian government health insurance database (Medicare), which includes all Australian citizens and permanent residents, and purposeful oversampling of people living outside urban areas. Women complete surveys every 2–3 years. The current study uses data from the cohort of women born between 1946 and 1951, who completed nine surveys between 1996 and 2019.

Women were included from the first survey (baseline, 1996) if they provided an age of menopause in any survey. Age of menopause was self-reported from Survey 2 onwards. The first reported age of menopause was included. Women were excluded if no age of menopause was provided, if a hysterectomy was performed prior to a reported age of menopause, or if age of menopause reported was greater than their current age. POI/EM was defined as menopause <45 years, and usual age menopause was defined as menopause ≥45 years. POI and EM women were combined into one group to optimize sample size. POI/EM was confirmed by ensuring absence of menses in the 12 months prior to the first survey, unless MHT or the combined oral contraceptive pill (COCP) was being used, as these questions were asked in the surveys. Bilateral oophorectomy was included within POI/EM if an age of surgery was provided. Given the age at first survey was 45–50 years, at baseline in 1996, all women with POI/EM had already gone through menopause.

Surveys were developed by the original study, and included demographic details, medical conditions, and self-reported biometry, which was used to calculate a BMI. Comorbidities known to influence osteoporosis were self-reported (hyperthyroidism, hyperparathyroidism, rheumatoid arthritis, malabsorption, inflammatory bowel disease, chronic liver disease, chronic kidney disease, diabetes, connective tissue disorders, any transplant). Residential location was defined using the Australian Statistical Geography Standard Remoteness Structure, categorized as major cities, inner regional, outer regional, remote and very remote areas, with remote and very remote locations combined due to small numbers (Australian Bureau of Statistics, July 2021–June 2026). Socioeconomic status was classified by the index of relative socioeconomic disadvantage (IRSD), which summarizes economic and social conditions of people by area and includes factors such as income and education, with lower values indicating greater disadvantage. Surveys are freely available online (Australian Longitudinal Study on Women’s Health, 2022).

The survey data have been linked to administrative health datasets, including the Pharmaceutical Benefits Scheme (PBS, Australian government-subsidized medications), Medicare Benefits Schedule (MBS, Australian government-subsidized medical services), and hospital separation data. Linked PBS data are available from 2002 onwards, MBS data are available from 1996 onwards, and availability of hospital separation data differs by state, with all states data included from 2007.

### Outcomes

The primary outcomes were to determine the proportion of women with POI/EM with osteoporosis and fractures and to identify predictors for developing osteoporosis/fractures. Secondary outcomes were the proportion of women with POI/EM undergoing osteoporosis investigation using dual X-ray absorptiometry (DXA), MHT use, and treatment with anti-osteoporosis medications. Secondary analyses aimed to identify predictors for these. Outcomes in women with POI/EM were identified and then compared with women with usual age menopause (age >45 years).

Osteoporosis diagnosis was collected from hospital separation data using ICD-9 and -10 codes for primary or secondary diagnoses (Supplementary Table S1) and was also self-reported in all surveys. The cumulative prevalence of osteoporosis is reported, as osteoporosis was primarily listed as a comorbidity in hospital separation data, and so incidence of osteoporosis could not be included.

Fractures were collected from hospital separation data from 1995 onwards, using ICD codes (Supplementary Table S1), and excluded fractures of the skull, hands, and feet. Fractures were also self-reported in all surveys except Survey 3 (2001). Self-reported fractures did not specify the location or number of fractures, and only reported incident fractures for the past 12 months. For this reason, fractures are reported as the cumulative prevalence of having had a fracture.

DXA rates were taken from MBS data, and self-reported in surveys from 2007 onwards. DXA rates are reported as the number who completed a DXA per survey period.

MHT use was collected from PBS data from 2002 onwards, and included transdermal or oral oestradiol, conjugated equine oestrogen ± medroxyprogesterone acetate, transdermal or oral oestradiol/norethisterone, oestrone, oestradiol/cyproterone, oestradiol hemihydrate, oestradiol/dydrogesterone, and the COCP, but excluding vaginal oestrogen preparations. MHT was also self-reported by participants from Survey 1 (1996) onwards. MHT treatment is reported as ‘current use of MHT’ (per survey period, use of any MHT or COCP since the previous survey); and ‘cumulative use of MHT’ (current or previous MHT at any time point, and COCP use from the Survey 1 onwards). We did not include self-reported COCP use prior to Survey 1, as the time of use was not provided, and the focus of this study was oestrogen for bone protection, rather than contraception.

Anti-osteoporosis medication was examined only in women who had either osteoporosis or a fracture and was available only from Survey 4 onwards. Treatment included all PBS-listed osteoporosis therapy (alendronate, risedronate, etidronate (together grouped as oral bisphosphonates), zoledronic acid, denosumab, raloxifene, strontium, teriparatide, and calcitriol). Treatment is reported as cumulative use at any time point. The number of prescriptions was used to determine duration of use and compliance. Compliance was defined as ≥80% medication possession index, except for denosumab and zoledronic acid. The antiresorptive effect of denosumab is rapidly reversed after missing or delaying a dose, and so compliance was defined as a subsequent dose within 7 months of the previous dose (Bone *et al.*, 2011). In contrast, zoledronic acid has a long half-life with prolonged efficacy, and so compliance was defined as subsequent dose within 18 months (Reid *et al.*, 2018).

### Statistical methods

Categorical variables were expressed as number (%), and categorical variables at baseline were compared between women with and without POI/EM using chi-square tests. Continuous variables were expressed as mean (SD) when normally distributed, and median (quartile 1, quartile 3) when not normally distributed. Continuous variables at baseline were compared between women with and without POI/EM using *t*-tests if normally distributed, and Mann–Whitney *U* test if not normally distributed. At each survey, the outcomes of osteoporosis, fractures, DXA, MHT, and anti-osteoporosis medications were compared between women with and without POI/EM using chi-square tests.

Generalized estimating equations for panel data were used to analyse predictive factors for the longitudinal outcomes of osteoporosis/fractures, DXA, MHT, and anti-osteoporosis medication in women with POI/EM. Independent variables included factors known to influence the risk of osteoporosis. The IRSD was included in multivariable models in place of education or marital status as it includes these factors. Interaction testing between residential location and IRSD was performed due to a theoretical relationship and found to be significant for the outcomes of osteoporosis, DXA and treatment, and so residential location only was included in these models. Univariable regression was performed, with variables retained at a significance level of *P* < 0.2, to form the final multivariable model. Bootstrapping was performed, with 100 repetitions at 95% sampling of the original dataset, to ensure robust results. All *P*-values were calculated from two-tailed tests of statistical significance, with *P* < 0.05 considered significant. Analyses were conducted using Stata software, v15 (StataCorp, Texas, USA).

### Ethical approval

Ethical approval was obtained from the University of Newcastle HREC (EC00144), the University of Queensland HREC (EC00456/7), the Australian Institute of Health and Welfare HREC (EC00103), the NSW Population and Health Services Research Ethics Committee (EC00410, this included a Victorian-specific module to address Victorian legislative requirements), the ACT Health HREC (EC00100), the Department of Health Western Australia HREC (EC00422), and Tasmanian Health and Medical HREC (EC00337).

## Results

**Figure 1.**
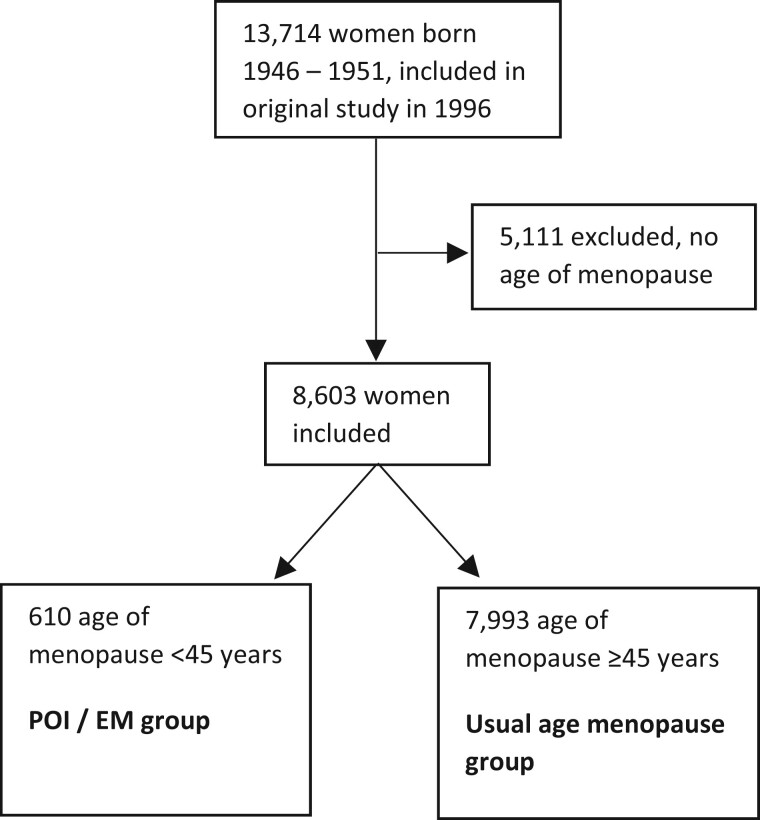
**Flowchart of participant inclusion.** POI: premature ovarian insufficiency; EM: early menopause.

Of the 13 714 women included in the original study in 1996, 8603 women reported an age of menopause and were included in the current study, including 610 (7.1%) with POI/EM (223 with POI) (Fig. 1). Baseline characteristics of women are listed in Table 1, the mean (SD) age at baseline was 47.6 (1.45) years, and the mean age at last follow-up was 67.8 (5.45) years. The mean (SD) age of menopause was 38.2 (7.95) and 51.3 (3.04) years in women with POI/EM and usual age menopause, respectively (*P* < 0.001).

**Table 1.** Baseline characteristics of included women.

		Premature ovarian insufficiency/early menopause n = 610	Usual age menopause n = 7993	Total n = 8603	*P*
Age (years; mean ± SD)		47.7 ± 1.4	47.6 ± 1.5	47.6 ± 1.5	**0.03**
BMI (kg/m^2^; mean ± SD)		25.5 ± 4.8	25.5 ± 4.9	25.5 ± 4.9	0.96
Education (n, %)	<High school	327 (54.5)	3583 (45.2)	3910 (45.8)	**<0.001**
	High school completion	109 (18.2)	1379 (17.4)	1488 (17.4)	
	Trade/apprentice/certificate/diploma	108 (18.0)	1614 (20.4)	1722 (20.2)	
	University/higher degree	56 (9.3)	1356 (17.1)	1412 (16.6)	
Marital status (n, %)	Partnered	467 (77.1)	6634 (83.4)	7101 (82.9)	**<0.001**
	Non-partnered	139 (22.9)	1323 (16.6)	1462 (17.1)	
Country of birth (n, %)	Australia	450 (74.9)	6052 (76.5)	6502 (76.4)	0.36
	Outside Australia	151 (25.1)	1858 (23.5)	2009 (23.6)	
IRSD (mean ± SD)		987.4 ± 94.5	1005 ± 88.2	1004.1 ± 88.8	**<0.001**
Residential location (n, %)	Major city	225 (36.9)	2920 (36.5)	3145 (36.6)	0.69
	Inner regional	227 (37.2)	3097 (38.8)	3324 (38.6)	
	Outer regional	128 (21.0)	1604 (20.1)	1732 (20.1)	
	Remote/very remote	30 (4.9)	372 (4.7)	402 (4.7)	
Smoking status (n, %)	Never	241 (40.8)	4262 (55.0)	4503 (54.0)	**<0.001**
	Former	156 (26.4)	2270 (29.3)	2426 (29.1)	
	Current	194 (32.8)	1213 (15.7)	1407 (16.9)	
Alcohol intake (n, %)	No	89 (14.8)	1064 (13.4)	1153 (13.5)	0.35
	Yes	514 (85.2)	6868 (86.6)	7382 (86.5)	
Menopause status (n, %)	Pre-menopausal	0	6886 (86.8)	6886 (80.6)	**<0.001**
	Post-menopausal	610 (100)	1045 (13.2)	1655 (19.4)	
Exogenous oestrogen (n, %)	Current use (MHT or COCP)	337 (55.3)	1457 (18.2)	1794 (20.9)	**<0.001**
	Past use (MHT only)	95 (15.6)	520 (6.5)	615 (7.2)	
	Never use	178 (29.2)	6016 (75.3)	6194 (72.0)	

Significant *P*-values are shown in bold.

COCP: combined oral contraceptive pill; IRSD: index of relative socioeconomic disadvantage; MHT: menopause hormone therapy (oral, transdermal, nasal).

Age, educational attainment, socioeconomic disadvantage, marital status, and smoking status differed between women with POI/EM and usual age menopause. At baseline, 432 (70.8%) of women with POI/EM were either currently using, or previously used MHT, compared to 1977 (24.7%) of women with usual age menopause. At baseline, 161 (1.9%) of women had bilateral oophorectomy, including 122 women with POI/EM, and 39 with usual age menopause; 149 women had a history of breast cancer (24 with POI/EM) and 337 had a history of endometrial, ovarian, or cervical cancer (39 with POI/EM). Comorbidities associated with an increased risk of osteoporosis, experienced over the 23-year follow-up period, varied between POI/EM and usual age menopause women (Table 2).

**Table 2.** Cumulative comorbidities during follow-up, by menopause group.

		Premature ovarian insufficiency/early menopause	Usual age menopause	Total	*P*
Hyperthyroidism		3 (0.5)	36 (0.5)	39 (0.5)	0.76
Hyperparathyroidism		1 (0.2)	6 (0.08)	7 (0.1)	0.40
Rheumatoid arthritis		85 (13.9)	751 (9.4)	836 (9.7)	**<0.001**
Malabsorption		1 (0.2)	25 (0.3)	26 (0.3)	1.00
Inflammatory bowel disease		0	37 (0.5)	37 (0.4)	0.11
Chronic liver disease		4 (0.7)	20 (0.3)	24 (0.3)	0.09
Chronic kidney disease		12 (2.0)	126 (1.6)	138 (1.6)	0.41
Diabetes		99 (16.2)	1086 (13.6)	1185 (13.8)	0.08
Connective tissue disease		12 (2.0)	79 (1.0)	91 (1.1)	**0.04**
Any transplant (solid organ and bone marrow)		6 (1.0)	9 (0.1)	15 (0.2)	**<0.001**
Any comorbidity		177 (29.0)	1849 (23.1)	2026 (23.6)	**0.001**
Number of comorbidities	1	133 (21.8)	1562 (19.5)	1695 (19.7)	**<0.001**
	2	43 (7.1)	257 (3.2)	300 (3.5)	
	3	1 (0.2)	28 (0.4)	29 (0.3)	
	4	0	2 (0.03)	2 (0.02)	

Data expressed as n (%). Significant *P*-values are shown in bold.

### Osteoporosis and fractures

Overall, 1936 (22.5%) of women had a diagnosis of osteoporosis during follow-up, including 191 (31.3%) of women with POI/EM and 1745 (21.8%) of women with usual age menopause. A fracture was recorded in 2015 (23.4%) women, including 190 (31.2%) of women with POI/EM and 1825 (22.8%) of women with usual age menopause. However, only 719 (35.7%) of those with a fracture had a known diagnosis of osteoporosis. Of those with POI/EM, 303 (49.7%) had either osteoporosis or a fracture, compared to 2929 (36.6%) of women with usual age menopause. Of the 842 fractures occurring in 738 women taken from hospital separation data, 500 of these were classified as major osteoporotic fractures (hip, spine, forearm, humerus), including 73 hip fractures in 69 women (10 women with POI/EM and 59 women with usual age menopause).

**Figure 2.**
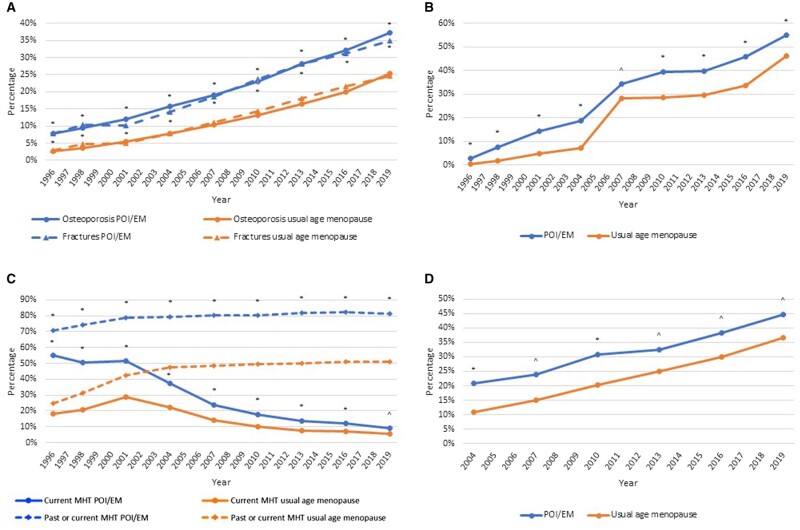
**Frequency of outcomes at each survey, by menopausal group.** (**A**) Cumulative frequency of osteoporosis and all fractures; (**B**) Dual X-ray absorptiometry use per survey period; (**C**) cumulative or current use of menopause hormone therapy; and (**D**) cumulative treatment with anti-osteoporosis medication in women with osteoporosis or fractures. Unadjusted frequency at each survey is compared by chi-square tests. *P < 0.001; ^*P* < 0.05. POI: premature ovarian insufficiency; EM: early menopause; MHT: menopause hormone therapy.

Osteoporosis and fracture prevalence was higher in women with POI/EM compared to women with usual age menopause at all surveys (Fig. 2A). Univariable and multivariable longitudinal analysis of the entire cohort found that women with POI/EM had significantly higher risk of osteoporosis (odds ratio [OR] 1.37, 95% CI 1.07–1.77) and fractures (OR 1.45, 95% CI 1.15–1.81), than women with usual age menopause (Supplementary Tables S2 and S3). In the entire cohort, increasing age, lower BMI, increasing number of comorbidities, and having had a DXA were also associated with greater likelihood of having osteoporosis, while current MHT use was protective (Supplementary Table S2). Increasing age and increasing number of comorbidities was associated with greater likelihood of having had a fracture, and current MHT use was protective (Supplementary Table S3). Bootstrapping did not alter results.

For women with POI/EM, univariable and multivariable longitudinal analysis indicated that increasing age was associated with an increased risk of osteoporosis/fracture (OR 1.09; 95% CI 1.08–1.11) (Table 3). MHT use prior to or at baseline, in 1996, but not having ever used MHT or current use of MHT, was protective for osteoporosis/fractures (OR 0.65; 0.45–0.96). BMI, smoking, alcohol, and the number of comorbidities were associated with osteoporosis/fractures on univariable analysis, but did not remain significant in the multivariable analysis. Bootstrapping did not alter results.

**Table 3.** Predictors of developing osteoporosis and/or fractures in women with premature ovarian insufficiency/early menopause.

Factor		Univariable model				Multivariable model			
		OR	95% CI		*P*-value	OR	95%		*P*-value
Age		1.10	1.08	1.11	**<0.001**	1.09	1.08	1.11	**<0.001**
BMI		1.07	1.04	1.10	**<0.001**	1.01	0.99	1.04	0.291
Smoking	Never	Ref							
	Former	0.94	0.75	1.17	0.550	0.94	0.72	1.23	0.664
	Current	0.48	0.35	0.66	**<0.001**	0.94	0.69	1.29	0.695
Alcohol	No	Ref							
	Yes	0.65	0.52	0.81	**<0.001**	0.85	0.68	1.06	0.147
Number of comorbidities		2.37	1.92	2.91	**<0.001**	1.12	0.88	1.42	0.373
Country of birth	Australia	Ref							
	Outside Australia	1.35	0.98	1.86	0.069	1.14	0.77	1.70	0.515
Residential location	Major city	Ref							
	Inner regional	0.85	0.66	1.10	0.212	1.06	0.82	1.36	0.657
	Outer regional	0.79	0.54	1.16	0.226	1.12	0.79	1.58	0.540
	Remote	0.70	0.45	1.09	0.117	1.57	0.96	2.58	0.074
IRSD		1.00	1.00	1.00	0.107	1.00	1.00	1.00	0.820
MHT use ever		1.41	1.03	1.94	**0.035**				
MHT use current		0.41	0.34	0.49	**<0.001**				
MHT prior to or at study entry		0.79	0.58	1.07	0.134	0.66	0.45	0.96	**0.032**

IRSD: index of relative socioeconomic disadvantage; MHT: menopause hormone therapy; OR: odds ratio.

Significant *P*-values are shown in bold.

### Investigation

Over the 23 years, 5341 (62.1%) of all women had at least one DXA (self-reported or MBS), including 421 (69.0%) of those with POI/EM and 4920 (61.6%) of those with usual age menopause. Of the 3886 who has an MBS DXA recorded, 1925 had a single DXA over the 23-year period, 839 had two DXA, and 1122 had >2 DXA done. Use of DXA was significantly higher in women with POI/EM compared with women with usual age menopause at all surveys (Fig. 2B), and longitudinal analysis also found POI/EM was associated with significantly higher likelihood of having a DXA (OR 1.64; 95% CI 1.42–1.90, Supplementary Table S4). Increasing age, lower BMI, increasing number of comorbidities, and previous fractures were also associated with a greater likelihood of having a DXA, while current smoking and living outside a major city were associated with reduced likelihood of DXA.

Of women with POI/EM, increasing age (OR 1.11; 1.10–1.12) and previous fracture (OR 1.80; 1.38, 2.34) were associated with greater likelihood of having a DXA (Supplementary Table S5). Living in an inner (OR 0.68; 0.53–0.88) or outer (0.63; 0.458–0.87) regional area, and current smoking (OR 0.60; 0.43–0.86) were associated with less likelihood of DXA. Increasing number of comorbidities increased the likelihood of DXA on univariable analysis, but after adjustment was no longer significant. Bootstrapping did not alter results.

### Menopause hormone therapy

MHT was used by 4488 (52.2%) of women before or during the study, including 474 (77.7%) of women with POI/EM and 4014 (50.2%) of women with usual age menopause. At baseline, 432/610 (70.8%) of women with POI/EM currently or previously used MHT, and of these, 22% used MHT for <1 year, 39% for 1–4 years, 28% for 5–10 years, and 6% for >10 years. Of women with osteoporosis or a fracture, 1820 (56.3%) had used MHT, including 232 (77.6%) of those with POI/EM and 1588 (54.2%) of those with usual age menopause. At all surveys, more women with POI/EM currently used MHT, and had ever used MHT, compared with women with usual age menopause (Fig. 2C). On longitudinal analysis, women with POI/EM had higher likelihood of having ever used MHT (OR 6.87, 95% CI 5.68–8.30, Supplementary Table S6). Increasing age and BMI were also associated with ever using MHT, while smoking, alcohol, number of comorbidities, and prevalent osteoporosis or fractures, was not.

In women with POI/EM, increasing age (OR 1.02; 1.01–1.02), and currently consuming alcohol (OR 1.17; 1.06–1.28), was associated with having ever used MHT (Supplementary Table S7). BMI, smoking, number of comorbidities, and prevalent osteoporosis or fracture were predictive of MHT use in univariable analysis but did not remain significant in the multivariable analysis. Bootstrapping did not alter results.

Types of MHT used, from PBS data from 2002 onwards, are in Supplementary Table S8. Of the 1180 women who used PBS MHT, the median (Q1, Q3) duration of use was 12.0 (4, 37) months, and 420 (35.6%) were compliant with therapy.

### Anti-osteoporosis medications

Analysis of treatment included 3215 participants with either osteoporosis or a fracture (297 with POI/EM, 2918 with usual age menopause), from Survey 4 (2004) onwards. A total of 1080 (33.6%) received anti-osteoporosis medications at some stage, including 116 (39.1%) of those with POI/EM and 964 (33.0%) of those with usual age menopause. Of the 1925 with OP, 938 (48.7%) received treatment, including 99/186 (53.2%) with POI/EM and 839/1739 (48.3%) with usual age menopause. Of those with a fracture, 596 (29.7%) received treatment, including 71/188 (37.8%) with POI/EM and 525/1818 (28.9%) with usual age menopause. At all surveys, women with POI/EM were significantly more likely to have used anti-osteoporosis medication, than women with usual age menopause (Fig. 2D), and longitudinal analysis confirmed women with POI/EM were more likely to use anti-osteoporosis medication (OR 1.50, 95% CI 1.14–1.98, Supplementary Table S9). Increasing age and lower BMI were also associated with using anti-osteoporosis medications, while current smoking was associated with lower likelihood of anti-osteoporosis medication use.

In the 299 women with POI/EM, increasing age (OR 1.07; 1.04–1.09) and lower BMI (OR 0.95; 0.92–0.98) were associated with greater likelihood of using anti-osteoporosis medications (Supplementary Table S10). There was no effect of smoking, alcohol, or the number of comorbidities. Bootstrapping did not alter results.

The types, duration, and compliance with anti-osteoporosis medications are listed in Supplementary Table S11. Compliance did not differ between women with and without POI/EM.

## Discussion

In this 23-year longitudinal study, by the age of 68 years, half of the women with POI/EM developed osteoporosis or a fracture. Increasing age was associated with an increased risk of osteoporosis/fractures in these women, whereas MHT use prior to or at study entry was protective. The risk of osteoporosis and fracture risk were 37% and 45% higher, respectively, in women with POI/EM compared with those with usual age menopause. Women with POI/EM were also more likely to have had a DXA, use MHT, and use anti-osteoporosis medications; however, the overall rates of use of the latter among women with osteoporosis or fractures was low at under 40%, highlighting a significant treatment gap in this population.

Our findings expand upon previous research of a subset of this cohort, which demonstrated that women with spontaneous menopause aged <40 years, and 41–45 years, had increased risk (OR 2.00 and 1.30, respectively) of incident osteoporosis over 6 years, compared with those undergoing menopause at 50–51 years (Xu *et al.*, 2020). Here, we provide longer follow-up duration, linked data and fracture data, and adjustment for confounding factors. Few other longitudinal studies include both women with POI and EM. A prospective longitudinal study of Swedish women over 34 years found 56% of women with POI/EM had osteoporosis by the age of 77 years, with an 83% increased risk of osteoporosis compared with women with later menopause (Svejme *et al.*, 2012). However, POI/EM was defined as menopause prior to the age of 47 years, rather than the conventional definition of prior to 45 years (The ESHRE Guideline Group on POI *et al.*, 2016). The current study provides convincing evidence for increased osteoporosis risk in women with POI/EM after accounting for other risk factors.

One-third of women with POI/EM experienced an incident fracture during this study. The current findings of increased fracture risk concur with a systematic review including 14 studies of women with POI/EM, which found a 36% increased risk of fracture compared with women with menopause >45 years, although there was heterogeneity between included studies, and possible differences between ethnic groups (Anagnostis *et al.*, 2019). Whether there is site-specific fracture risk remains unclear. A longitudinal study including 49 000 women with POI/EM who had never used MHT found little if any increased risk for hip fracture (Banks *et al.*, 2009). Mixed findings are reported for vertebral fractures, with no increase in self-reported vertebral fractures in Canadian and Japanese cohorts with POI/EM (Papaioannou *et al.*, 2005; Shimizu *et al.*, 2018). However, a study of Dutch women found a 90% increased risk for vertebral fractures confirmed on spinal X-rays in women with POI/EM compared to those with menopause age >50 years (Klift *et al.*, 2004). Unfortunately, fracture site was not collected in ALSWH survey data, and the low number of fractures for each site recorded in linked hospital data prevented further exploration. Ongoing follow-up of the current cohort may enable such comparisons in the future.

In women with POI/EM, increasing age was associated with an increased risk of osteoporosis or fracture. This is consistent with a previous review, which also found no effect of BMI, but which did not examine other potential risk factors (Anagnostis *et al.*, 2019). Studies limited to women with spontaneous POI, reported increased risk of low BMD associated with earlier onset of menstrual irregularity, delay in diagnosis and commencement of MHT, poor adherence to MHT, low vitamin D, low body weight, low calcium intake, lack of regular exercise, and increasing age (Leite-Silva *et al.*, 2009; Popat *et al.*, 2009). However, these cross-sectional studies only examined women at a younger age (mean age ∼30 years), and risk factors at only one time point. A strength of the current study was that risk factors were assessed at multiple time points, which takes into consideration changes in smoking status, body weight, and other dynamic risk factors over time.

Using MHT ever, at the start of the study (aged 45–50 years), was protective for osteoporosis/fractures, whereas having ever used MHT later was not protective. This highlights the importance of early treatment with MHT in women who undergo POI/EM. International guidelines recommend MHT in women with POI/EM, without contraindications, until the age of usual menopause, to prevent osteoporosis (The ESHRE Guideline Group on POI *et al.*, 2016; The North American Menopause Society, 2022). Delay in diagnosis/treatment, non-adherence to MHT, dose, and type of MHT appear to influence the BMD response to MHT (Burgos *et al.*, 2017; Gonçalves *et al.*, 2022; Samad *et al.*, 2022; Costa *et al.*, 2023), and there is as yet no definite evidence for fracture reduction (Gonçalves *et al.*, 2022). Although our findings suggest a protective effect of early MHT on fractures, being observational, we cannot determine causality. At study entry, 70% of women with POI/EM had previously or currently used MHT. Of concern is that 20% of women with POI/EM did not receive appropriate MHT (after excluding women with possible oestrogen-responsive cancers). Sub-optimal MHT use by women with POI/EM has been reported in other studies with the proportion of women using MHT varying between 22 and 50% (Shea *et al.*, 2021; Svejme *et al.*, 2012). Additionally, 30–40% of women with POI are non-adherent to MHT, due to a perceived lack of benefit, side effects, or fears of breast cancer (Popat *et al.*, 2009; Bachelot *et al.*, 2016). Unfortunately, due to the nature of the current study, it was not possible to examine the type, dose, adherence, and duration of MHT in earlier life, limiting the ability to examine these factors.

DXA is recommended for all women with POI at baseline, with follow-up imaging dependent on BMD (Kiriakova *et al.*, 2019; The ESHRE Guideline Group on POI *et al.*, 2016). In our cohort, during the follow-up period, 69% of women with POI/EM had one or more DXA performed, and women with POI/EM were significantly more likely to have a DXA than women with usual age menopause. DXA utilization was similar to that in cross-sectional studies of Australian women with POI (Gibson-Helm *et al.*, 2014; Jones *et al.*, 2020). However, DXA performed prior to study entry or at diagnosis of POI/EM was not captured in our study, and thus the total number of women with POI/EM who completed screening at any time is likely to be higher. Although women with POI/EM were more likely than women with usual age menopause to receive osteoporosis treatment, management of osteoporosis overall was sub-optimal, with <40% of women with osteoporosis/fractures receiving an anti-osteoporosis medication at any stage. This reflects a known global gap in osteoporosis care with low rates of antiresorptive use (20–30%) and failure to prevent re-fractures, and highlights an important practice point for clinicians (Kanis *et al.*, 2021). There are no specific guidelines for using antiresorptive agents in women with POI/EM, although studies in younger women have demonstrated increased BMD with bisphosphonate therapy (Tauchmanova *et al.*, 2006; Shapiro *et al.*, 2011; Kiriakova *et al.*, 2019).

This study has several strengths. It is the largest study of bone health in Australian women with POI/EM, one of few longitudinal studies assessing osteoporosis and fractures in this population, and the first to report on longitudinal risk factors, investigation, and anti-osteoporosis medication. Although POI/EM was self-reported, it was validated by ensuring all women with POI/EM had amenorrhoea at study entry. Outcomes are both self-reported and derived from administrative records. There are also several limitations to our study. Survey data were self-reported, and given data collection commenced from age 45 to 50 years, limited data were available on earlier risk factors, fractures, screening, treatment, or MHT use. We were unable to include additional risk/protective factors such as vitamin D use, calcium intake, or exercise. Although the prevalence of POI/EM was similar to that reported previously (Golezar *et al.*, 2019), we were unable to distinguish between different causes of POI/EM. Self-reported fractures only included those in the prior 12 months, rather than the 3-year survey period included for hospital separation data fractures, and vertebral fractures will be underestimated without performing routine X-rays. Linked administrative databases were not available for the full study period, with some states’ hospital separation data not available for earlier surveys, and PBS medications were only available from 2002. Compliance estimations assumed that women took all medications dispensed. Finally, the cohort had greater Australian-born women, with higher educational levels, than the broader Australian population (Dobson *et al.*, 2015).

In this large prospective cohort of Australian women over 23 years, 7% experienced POI/EM, and half of women with POI/EM had osteoporosis or a fracture by the age of 68 years. In women with POI/EM, MHT use prior to or at the age of 45–50 years was protective for osteoporosis/fractures, highlighting the importance of early treatment in managing women with POI/EM. Although DXA utilization was high, one-fifth of women did not receive appropriate MHT and use of anti-osteoporosis medications were low, highlighting a significant practice gap in preventing future fractures.

## Supplementary Material

deae037_Supplementary_Table_S1

deae037_Supplementary_Table_S2

deae037_Supplementary_Table_S3

deae037_Supplementary_Table_S4

deae037_Supplementary_Table_S5

deae037_Supplementary_Table_S6

deae037_Supplementary_Table_S7

deae037_Supplementary_Table_S8

deae037_Supplementary_Table_S9

deae037_Supplementary_Table_S10

deae037_Supplementary_Table_S11

## Data Availability

ALSWH survey data are owned by the Australian Government Department of Health and Aged Care and due to the personal nature of the data collected, release by ALSWH is subject to strict contractual and ethical restrictions. Ethical review of ALSWH is by the Human Research Ethics Committees at The University of Queensland and The University of Newcastle. De-identified data are available to collaborating researchers where a formal request to make use of the material has been approved by the ALSWH Data Access Committee and by relevant data custodians (if applicable). The committee is receptive of requests for datasets required to replicate results. Information on applying for ALSWH data is available from https://alswh.org.au/for-data-users/applying-for-data/ The Centre for Health Record Linkage (CHeReL), NSW Ministry of Health and ACT Health, for the NSW Admitted Patients Collection; and the ACT Admitted Patient Care Collection. Queensland Health as the source for Queensland Hospital Admitted Patient Data Collection; and the Statistical Analysis and Linkage Unit (Queensland Health) for the provision of data linkage. The Department of Health Western Australia, including the Data Linkage Branch, and the WA Hospital Morbidity Data Collection. SA NT Datalink, SA Health, and Northern Territory Department of Health, for the SA Public Hospital Separations, and NT Public Hospital Inpatient Activity Data Collections. The Department of Health Tasmania, and the Tasmanian Data Linkage Unit, for the Public Hospital Admitted Patient Episodes Data Collection. Victorian Department of Health as the source of the Victorian Admitted Episodes Dataset; and the Centre for Victorian Data Linkage (Victorian Department of Health) for the provision of data linkage.
