# Supplementary material for: Bone health in women with premature ovarian insufficiency/early menopause: a 23-year longitudinal analysis
Source: Hum Reprod. 2024 Feb 23;39(5):1013–22. doi: 10.1093/humrep/deae037 (PMC11063537; doi:10.1093/humrep/deae037)
Supplement: deae037_Supplementary_Table_S1 [file deae037_supplementary_table_s1.pdf]

OXFORD
human reproduction

Human Reproduction, 2024, 1–4
https://doi.org/10.1093/humrep/deae037
Supplementary Data

Supplementary Table S1. Hospital admissions data coding of osteoporosis and fractures, based on ICD-10-AM.

Hip/femur fracture	Vertebral fracture	Upper arm/shoulder fracture	Forearm fracture	Pelvis fracture	Lower leg fracture	Ankle fracture	Clavicle/scapula fracture	Rib/sternum fracture
S72.00	M48.50	M80.01	M80.13	M80.05	M80.06	M80.07	S42.10	S22.2
S72.01	M48.51	M80.02	M80.23	M80.15	M80.16	M80.17	S42.11	S22.31
S72.02	M48.52	M80.11	M80.33	M80.25	M80.26	M80.27	S42.12	S22.32
S72.03	M48.53	M80.12	M80.43	M80.35	M80.36	M80.37	S42.13	S22.40
S72.04	M48.54	M80.21	M80.53	M80.45	M80.46	M80.47	S42.14	S22.41
S72.05	M48.55	M80.22	M80.83	M80.55	M80.56	M80.57	S42.19	S22.42
S72.08	M48.56	M80.31	M80.93	M80.85	M80.86	M80.87	S42.00	S22.43
S72.10	M48.57	M80.32	M84.33	M80.95	M80.96	M80.97	S42.01	S22.44
S72.11	M48.58	M80.41	M84.43	M84.35	M84.36	M84.37	S42.02	
S72.2	M48.59	M80.42	S51.81	M84.45	M84.46	M84.47	S42.03	
S72.3	M48.40	M80.51	S52.00	S32.2	S81.81	S82.5	S42.09	
S72.40	M48.41	M80.52	S52.01	S32.3	S82.0	S82.6		
S72.41	M48.42	M80.81	S52.02	S32.4	S82.11	S82.81		
S72.42	M48.43	M80.82	S52.09	S32.5	S82.18	S82.82		
S72.43	M48.44	M80.91	S52.20	S32.7	S82.21			
S72.44	M48.45	M80.92	S52.21	S32.81	S82.28			
S72.7	M48.46	M84.31	S52.4	S32.83	S82.31			
S72.8	M48.47	M84.32	S52.6	S32.89	S82.38			
S72.9	M48.48	M84.41	S52.10		S82.40			
S71.81	M48.49	M84.42	S52.11		S82.41			
	S11.81	S41.81	S52.12		S82.42			
	S12.0	S42.20	S52.19		S82.49			
	S12.1	S42.21	S52.30		S82.7			
	S12.21	S42.22	S52.31		S82.88			
	S12.22	S42.23	S52.50		S82.9			
	S12.23	S42.24	S52.51					
	S12.24	S42.29	S52.52					
	S12.25	S42.3	S52.53					
	S12.7	S42.40	S52.59					
	S12.8	S42.41	S52.7					
	S12.9	S42.42	S52.8					
	S21.81	S42.43	S52.9					
	S22.00	S42.44						
	S22.01	S42.45						
	S22.02	S42.49						
	S22.03	S42.7						
	S22.04	S42.8						
	S22.05							
	S22.06							
	S22.1							
	S32.00							
	S32.01							
	S32.02							
	S32.03							
	S32.04							
	S32.05							
	S32.82							
	S32.1							

Unspecified fracture	Osteopor-osis diagnosis
S22.8	M80.00
S22.9	M80.01
S31.81	M80.02
M80.00	M80.03
M80.10	M80.04
M80.20	M80.05
M80.30	M80.06
M80.40	M80.07
M80.50	M80.08
M80.80	M80.09
M80.90	M80.10
M84.30	M80.11
M84.40	M80.12

(continued)

Supplementary Table S1. Continued

Unspecified fracture	Osteopor-osis diagnosis
	M80.13
	M80.14
	M80.15
	M80.16
	M80.17
	M80.18
	M80.19
	M80.20
	M80.21
	M80.22
	M80.23
	M80.24
	M80.25
	M80.26
	M80.27
	M80.28
	M80.29
	M80.30
	M80.31
	M80.32
	M80.33
	M80.34
	M80.35
	M80.36
	M80.37
	M80.38
	M80.39
	M80.40
	M80.41
	M80.42
	M80.43
	M80.44
	M80.45
	M80.46
	M80.47
	M80.48
	M80.49
	M80.50
	M80.51
	M80.52
	M80.53
	M80.54
	M80.55
	M80.56
	M80.57
	M80.58
	M80.59
	M80.80
	M80.81
	M80.82
	M80.83
	M80.84
	M80.85
	M80.86
	M80.87
	M80.88
	M80.89
	M80.90
	M80.91
	M80.92
	M80.93
	M80.94
	M80.95
	M80.96
	M80.97
	M80.98
	M80.99
	M81.00
	M81.01
	M81.02
	M81.03
	M81.04

(continued)

Supplementary Table S1. Continued

Unspecified fracture	Osteopor-osis diagnosis
	M81.05
	M81.06
	M81.07
	M81.08
	M81.09
	M81.10
	M81.11
	M81.12
	M81.13
	M81.14
	M81.15
	M81.16
	M81.17
	M81.18
	M81.19
	M81.20
	M81.21
	M81.22
	M81.23
	M81.24
	M81.25
	M81.26
	M81.27
	M81.28
	M81.29
	M81.30
	M81.31
	M81.32
	M81.33
	M81.34
	M81.35
	M81.36
	M81.37
	M81.38
	M81.39
	M81.40
	M81.41
	M81.42
	M81.43
	M81.44
	M81.45
	M81.46
	M81.47
	M81.48
	M81.49
	M81.50
	M81.51
	M81.52
	M81.53
	M81.54
	M81.55
	M81.56
	M81.57
	M81.58
	M81.59
	M81.80
	M81.81
	M81.82
	M81.83
	M81.84
	M81.85
	M81.86
	M81.87
	M81.88
	M81.89
	M81.90
	M81.91
	M81.92
	M81.93
	M81.94
	M81.95
	M81.96

(continued)

Supplementary Table S1. Continued

Unspecified fracture	Osteopor-osis diagnosis
	M81.97
	M81.98
	M81.99
	M81.60
	M81.61
	M81.62
	M81.63
	M81.64
	M81.65
	M81.66
	M81.67
	M81.68
	M81.69
	U86.4
	U86.40
	M82.00
	M82.01
	M82.02
	M82.03
	M82.04
	M82.05
	M82.06
	M82.07
	M82.08
	M82.09
	M82.10
	M82.11
	M82.12
	M82.13
	M82.14
	M82.15
	M82.16
	M82.17
	M82.18
	M82.19
	M82.80
	M82.81
	M82.82
	M82.83
	M82.84
	M82.85
	M82.86
	M82.87
	M82.88
	M82.89

OXFORD
human reproduction

Human Reproduction, 2024, 1–1
https://doi.org/10.1093/humrep/deae037
Supplementary Data

Supplementary Table S2. Predictors of osteoporosis in entire cohort, longitudinal analysis.

Factor		Univariable model				Multivariable model			
		OR	95% CI		P-value	OR	95% CI		P-value
POI/EM		1.92	1.61	2.30	**<0.001**	1.37	1.07	1.77	**0.02**
Age		1.01	1.10	1.10	**<0.001**	1.06	1.06	1.06	**<0.001**
BMI		1.04	1.04	1.05	**<0.001**	0.96	0.95	0.97	**<0.001**
Smoking	Never	Ref							
	Former	0.92	0.84	1.00	**0.05**	1.03	0.92	1.14	0.64
	Current	0.44	0.37	0.53	**<0.001**	1.01	0.85	1.18	0.96
Alcohol	No								
	Yes	0.74	0.67	0.81	**<0.001**	0.92	0.83	1.02	0.12
Number of comorbidities		2.52	2.35	2.71	**<0.001**	1.28	1.15	1.42	**<0.001**
Country of birth	Australia	Ref							
	Outside Australia	1.11	0.98	1.26	0.09	1.07	0.90	1.27	0.43
Residential location	Major city	Ref							
	Inner regional	0.89	0.81	0.99	**0.03**	1.09	0.98	1.21	0.12
	Outer regional	0.678	0.59	0.78	**<0.001**	1.07	0.93	1.23	0.37
	Remote	0.41	0.30	0.55	**<0001**	1.02	0.81	1.29	0.87
IRSD		1.00	1.00	1.00	0.12				
Previous DXA		6.48	5.95	7.05	**<0.001**	3.48	3.18	3.81	**<0.001**
Current MHT		0.58	0.53	0.64	**<0.001**	1.10	1.01	1.19	**0.04**

COCP: combined oral contraceptive pill; DXA: dual-energy X-ray absorptiometry; IRSD: index of relative socioeconomic disadvantage; MHT: current menopause hormone therapy/COCP use; OR: odds ratio; POI/EM: premature ovarian insufficiency/early menopause.
Significant *P*-values are given in bold.

OXFORD
human reproduction

Human Reproduction, 2024, 1–1
https://doi.org/10.1093/humrep/deae037
Supplementary Data

Supplementary Table S3. Predictors of fractures in entire cohort, longitudinal analysis.

Factor		Univariable model				Multivariable model			
		OR	95% CI		P-value	OR	95% CI		P-value
POI/EM		1.74	1.45	2.08	**<0.001**	1.45	1.15	1.81	**0.001**
Age		1.10	1.09	1.10	**<0.001**	1.10	1.10	1.11	**<0.001**
BMI		1.06	1.06	1.07	**<0.001**	1.01	1.00	1.02	0.11
Smoking	Never	Ref							
	Former	0.87	0.80	0.95	**0.001**	1.03	0.93	1.13	0.59
	Current	0.45	0.37	0.53	**<0.001**	1.11	0.97	1.28	0.13
Alcohol	No	Ref							
	Yes	0.75	0.69	0.82	**<0.001**	0.99	0.90	1.09	0.89
Number of comorbidities		2.23	2.09	2.39	**<0.001**	1.12	1.03	1.23	**0.011**
Country of birth	Australia	Ref							
	Outside Australia	1.04	0.92	1.17	0.54				
Residential location	Major city	Ref							
	Inner regional	0.90	0.81	0.99	**0.04**	0.96	0.87	1.05	0.37
	Outer regional	0.81	0.71	0.92	**0.001**	0.99	0.88	1.13	0.92
	Remote	0.57	0.44	0.75	**<0.001**	1.08	0.86	1.34	0.51
IRSD		1.00	1.00	1.00	**0.001**	1.00	1.00	1.00	0.87
Current MHT		0.47	0.43	0.52	**<0.001**	0.88	0.82	0.95	**0.002**

IRSD: index of relative socioeconomic disadvantage; MHT: current menopause hormone therapy/combined oral contraceptive pill use; OR: odds ratio; POI/EM: premature ovarian insufficiency/early menopause.
Significant P-values are given in bold.

OXFORD
human reproduction
Human Reproduction, 2024, 1–1
https://doi.org/10.1093/humrep/deae037
Supplementary Data

Supplementary Table S4. Predictors of having a dual-energy X-ray absorptiometry, longitudinal analysis.

Factor		Univariable model				Multivariable model			
		OR	95% CI		P-value	OR	95% CI		P-value
POI/EM		1.56	1.40	1.73	**<0.001**	1.64	1.42	1.90	**<0.001**
Age		1.14	1.14	1.15	**<0.001**	1.15	1.15	1.16	**<0.001**
BMI		1.01	1.01	1.02	**<0.001**	0.96	0.96	0.97	**<0.001**
Smoking	Never	Ref							
	Former	0.98	0.92	1.04	0.47	0.99	0.92	1.06	0.72
	Current	0.35	0.31	0.40	**<0.001**	0.66	0.58	0.75	**<0.001**
Alcohol	No	Ref							
	Yes	0.91	0.85	0.98	**0.011**	1.09	0.99	1.19	0.07
Number of comorbidities		2.27	2.14	2.41	**<0.001**	1.14	1.05	1.23	**0.002**
Country of birth	Australia	Ref							
	Outside Australia	1.01	0.95	1.08	0.75				
Residential location	Major city	Ref							
	Inner regional	0.73	0.69	0.78	**<0.001**	0.76	0.70	0.82	**<0.001**
	Outer regional	0.60	0.55	0.65	**<0.001**	0.73	0.66	0.81	**<0.001**
	Remote	0.47	0.39	0.57	**<0.001**	0.92	0.76	1.11	0.39
IRSD		1.00	1.00	1.00	**<0.001**				
Current or past fracture		4.11	3.84	4.40	**<0.001**	1.98	1.81	2.16	**<0.001**

DXA: dual-energy X-ray absorptiometry; IRSD: index of relative socioeconomic disadvantage; OR: odds ratio; POI/EM: premature ovarian insufficiency/early menopause.
Significant P-values are given in bold.

human reproduction

Human Reproduction, 2024, 1–1
https://doi.org/10.1093/humrep/deae037
Supplementary Data

Supplementary Table S5. Predictors of dual-energy X-ray absorptiometry in women with premature ovarian insufficiency/early menopause, longitudinal analysis.

Factor		Univariable model				Multivariable model			
		OR	95% CI		P-value	OR	95%		P-value
Age		1.12	1.11	1.33	**<0.001**	1.11	1.10	1.12	**<0.001**
BMI		0.99	0.97	1.01	0.40				
Smoking	Never	Ref							
	Former	0.94	0.76	1.16	0.556	0.90	0.70	1.16	0.41
	Current	0.32	0.22	0.46	**<0.001**	0.60	0.43	0.86	**0.005**
Alcohol	No								
	Yes	0.85	0.65	1.11	0.236				
Number of comorbidities		2.37	1.97	2.85	**<0.001**	1.13	0.92	1.40	0.23
Country of birth	Australia	Ref							
	Outside Australia	1.14	0.91	1.44	0.253				
Residential location	Major city	Ref							
	Inner regional	0.70	0.55	0.89	**0.004**	0.68	0.53	0.88	**0.003**
	Outer regional	0.58	0.42	0.80	**0.001**	0.63	0.46	0.87	**0.004**
	Remote	0.35	0.16	0.78	**0.010**	0.55	0.29	1.05	0.07
IRSD		1.00	1.00	1.00	0.45				
Current or past fracture		3.489	2.79	4.37	**<0.001**	1.80	1.38	2.34	**<0.001**

IRSD: index of relative socioeconomic disadvantage; OR: odds ratio.
Significant *P*-values are shown in bold.

OXFORD
human reproduction
Human Reproduction, 2024, 1–1
https://doi.org/10.1093/humrep/deae037
Supplementary Data

Supplementary Table S6. Predictors of having ever used menopause hormone therapy, longitudinal analysis.

Factor		Univariable model				Multivariable model			
		OR	95% CI		P-value	OR	95% CI		P-value
POI/EM		4.25	3.54	5.11	**<0.001**	6.87	5.68	8.30	**<0.001**
Age		1.04	1.04	1.04	**<0.001**	1.04	1.04	1.05	**<0.001**
BMI		1.06	1.05	1.06	**<0.001**	1.01	1.01	1.02	**<0.001**
Smoking	Never	Ref							
	Former	0.88	0.84	0.92	**<0.001**	1.00	0.96	1.06	0.88
	Current	0.65	0.61	0.70	**<0.001**	1.02	0.95	1.10	0.61
Alcohol	No	Ref							
	Yes	0.89	0.85	0.93	**<0.001**	1.00	0.96	1.05	0.90
Number of comorbidities		1.47	1.41	1.53	**<0.001**	0.98	0.93	1.02	0.27
Country of birth	Australia	Ref							
	Outside Australia	1.01	0.92	1.10	0.91				
Residential location	Major city	Ref							
	Inner regional	0.92	0.87	0.96	**<0.001**	0.99	0.94	1.03	0.54
	Outer regional	0.82	0.77	0.88	**<0.001**	0.98	0.91	1.04	0.49
	Remote	0.70	0.62	0.79	**<0.001**	0.99	0.88	1.11	0.81
IRSD		1.00	1.00	1.00	0.31				
Prevalent osteoporosis or fractures		1.68	1.61	1.75	**<0.001**	1.01	0.96	1.06	0.63

IRSD: index of relative socioeconomic disadvantage; OR: odds ratio; POI/EM: premature ovarian insufficiency/early menopause.
Significant P-values are given in bold.

Human Reproduction, 2024, 1–1
https://doi.org/10.1093/humrep/deae037
Supplementary Data

Supplementary Table S7. Predictors of past or current menopause hormone therapy use in women with premature ovarian insufficiency/early menopause, longitudinal analysis.

Factor		Univariable model				Multivariable model			
		OR	95% CI		P-value	OR	95% CI		P-value
Age		1.02	1.01	1.02	**<0.001**	1.02	1.01	1.02	**<0.001**
BMI		1.02	1.00	1.03	**0.02**	1.00	0.99	1.02	0.72
Smoking	Never	Ref							
	Former	0.88	0.75	1.03	0.10	0.88	0.74	1.04	0.14
	Current	0.71	0.57	0.88	**0.001**	0.81	0.65	1.02	0.07
Alcohol	No	Ref							
	Yes	1.08	0.99	1.18	0.08	1.17	1.06	1.28	**0.001**
Number of comorbidities		1.12	1.01	1.24	**0.03**	0.99	0.88	1.11	0.85
Country of birth	Australia	Ref							
	Outside Australia	0.74	0.49	1.12	0.15	0.78	0.53	1.21	0.29
Aria	Major city	Ref							
	Inner regional	0.95	0.86	1.05	0.28	0.98	0.88	1.09	0.69
	Outer regional	0.86	0.74	0.99	0.04	0.92	0.78	1.07	0.28
	Remote	1.12	0.74	1.71	0.59	1.31	0.84	2.05	0.24
IRSD		1.00	1.00	1.00	0.49				
Prevalent osteoporosis or fracture		1.17	1.07	1.29	**0.001**	0.95	0.85	1.07	0.40

IRSD: index of relative socioeconomic disadvantage; OR: odds ratio.
Significant *P*-values are given in bold.

OXFORD

human reproduction

Human Reproduction, 2024, 1–1
https://doi.org/10.1093/humrep/deae037
Supplementary Data

Supplementary Table S8. Types of Pharmaceutical Benefits Scheme-derived menopause hormone therapy used.

	Early menopause (n = 610)	Usual age menopause (n = 7993)	Total (n = 8603)
Transdermal oestradiol	50 (8.2)	200 (2.5)	250 (2.9)
Oral oestradiol	19 (3.1)	99 (1.2)	118 (1.4)
Conjugated equine oestrogen	44 (7.2)	225 (2.8)	269 (3.1)
Conjugated equine oestrogen + medroxyprogesterone acetate	25 (4.1)	356 (4.5)	381 (4.4)
Transdermal oestradiol + norethisterone	11 (1.8)	205 (2.6)	216 (2.5)
Oral oestradiol + norethisterone	11 (1.8)	256 (3.2)	267 (3.1)
Oestrone	19 (3.1)	46 (0.6)	65 (0.8)
Oestradiol + cyproterone	0 (0.0)	2 (0.03)	2 (0.02)
Oestradiol hemihydrate	1 (0.2)	8 (0.1)	9 (0.1)
Oestradiol + dydrogesterone	2 (0.3)	20 (0.3)	22 (0.3)
Combined oral contraceptive pill	2 (0.3)	40 (0.5)	42 (0.5)

Data are from 2002 onwards. All values are n (%).

OXFORD

human reproduction

Human Reproduction, 2024, 1–1

https://doi.org/10.1093/humrep/deae037

Supplementary Data

Supplementary Table S9. Predictors of osteoporosis treatment in women with osteoporosis/fracture, longitudinal analysis.

Factor		Univariable model				Multivariable model			
		OR	95% CI		P-value	OR	95% CI		P-value
POI/EM		1.44	1.13	1.83	**0.003**	1.50	1.14	1.98	**0.004**
Age		1.09	1.08	1.10	**<0.001**	1.09	1.08	1.10	**<0.001**
BMI		0.96	0.95	0.98	**<0.001**	0.95	0.94	0.97	**<0.001**
Smoking	Never	Ref							
	Former	0.89	0.80	1.01	0.06	0.98	0.86	1.11	0.73
	Current	0.55	0.43	0.69	**<0.001**	0.79	0.62	1.00	**0.05**
Alcohol	No	Ref							
	Yes	0.87	0.78	0.98	**0.03**	1.06	0.92	1.21	0.42
Number of comorbidities		1.35	1.24	1.47	**<0.001**	1.04	0.93	1.16	0.51
Country of birth	Australia	Ref							
	Outside Australia	1.02	0.86	1.20	0.84				
Residential location	Major city	Ref							
	Inner regional	0.95	0.83	1.09	0.45	0.92	0.80	1.06	0.24
	Outer regional	0.86	0.73	1.01	0.06	0.91	0.76	1.08	0.29
	Remote	0.67	0.46	0.96	**0.03**	0.84	0.58	1.22	0.36
IRSD		1.00	1.00	1.00	**0.02**				

IRSD: index of relative socioeconomic disadvantage; OR: odds ratio; POI/EM: premature ovarian insufficiency/early menopause.
Significant P-values are shown in bold.

Human Reproduction, 2024, 1–1
https://doi.org/10.1093/humrep/deae037
Supplementary Data

Supplementary Table S10. Predictors of past or current use of anti-osteoporosis medications in women with premature ovarian insufficiency/early menopause and osteoporosis or a fracture.

Factor		Univariable model				Multivariable model			
		OR	95% CI		P-value	OR	95% CI		P-value
Age		1.07	1.05	1.09	**<0.001**	1.07	1.04	1.09	**<0.001**
BMI		0.96	0.93	0.99	**0.003**	0.95	0.92	0.98	**0.001**
Smoking	Never	Ref							
	Former	1.04	0.71	1.52	0.84	1.05	0.67	1.65	0.83
	Current	0.67	0.43	1.06	0.08	0.78	0.47	1.28	0.32
Alcohol	No	Ref							
	Yes	0.82	0.65	1.02	0.08	0.93	0.70	1.23	0.60
Number of comorbidities		1.32	1.03	1.72	**0.04**	1.05	0.78	1.41	0.76
Country of birth	Australia	Ref							
	Outside Australia	0.87	0.52	1.47	0.61				
Aria	Major city	Ref							
	Inner regional	1.01	0.68	1.51	0.95				
	Outer regional	0.95	0.68	1.31	0.73				
	Remote	0.87	0.58	1.30	0.49				
IRSD		1.00	1.00	1.00	0.61				

IRSD: index of relative socioeconomic disadvantage; OR: odds ratio.
Significant P-values are shown in bold.

OXFORD
human reproduction
Human Reproduction, 2024, 1–1
https://doi.org/10.1093/humrep/deae037
Supplementary Data

Supplementary Table S11. Types of bone-specific treatments used in women with osteoporosis or fracture.

	Early menopause N = 297			No early menopause N = 2918			Total N = 3215		
	Number (%)	Duration (median, Q1, Q3)	Compliant (N, %)	Number (%)	Duration (median, Q1, Q3)	Compliant (%)	Number (%)	Duration (median, Q1, Q3)	Compliant (N, %)
PO BP	82 (27.6)	27 (7, 69)	43 (52.4)	570 (19.5)	31 (7, 61)	306 (53.7)	652 (20.3)	30 (7, 62)	349 (53.5)
Denosumab	64 (21.6)	24 (12, 54)	54 (84.4)	546 (18.7)	24 (12, 42)	421 (77.1)	610 (19.0)	24 (12, 48)	475 (77.9)
Zoledronic acid	12 (4.0)	48 (42, 60)	12 (100)	75 (2.6)	36 (12, 48)	75 (100)	87 (2.7)	36 (12, 60)	87 (100)
Teriparatide	1 (0.3)	23 (23, 23)	1 (100)	8 (0.3)	22 (18, 24.5)	7 (87.5)	9 (0.3)	22 (20, 24)	8 (88.9)
Raloxifene	12 (4.0)	36 (18.5, 53.5)	8 (66.7)	39 (1.3)	20 (6, 62)	22 (56.4)	51 (1.6)	29 (6, 61)	30 (58.8)
Strontium	11 (3.7)	12 (6, 32)	6 (54.6)	101 (3.5)	9 (3, 26)	36 (35.6)	112 (3.5)	9 (3, 26)	42 (37.5)
Calcitriol	7 (2.4)	12.8 (3.2, 32)	2 (28.6)	37 (1.3)	19.2 (11.2, 65.6)	15 (40.5)	44 (1.4)	19.2 (10.4, 53.6)	17 (38.6)

PO BP: oral bisphosphonates, including alendronate, risedronate, and etidronate.
